# Correction: Topchu et al. PIP4K2B Protein Regulation by NSD1 in HPV-Negative Head and Neck Squamous Cell Carcinoma. *Cancers* 2024, *16*, 1180

**DOI:** 10.3390/cancers17223650

**Published:** 2025-11-14

**Authors:** Iuliia Topchu, Igor Bychkov, Ekaterina Roshchina, Petr Makhov, Yanis Boumber

**Affiliations:** 1 Robert H. Lurie Comprehensive Cancer Center, Feinberg School of Medicine, Division of Hematology/Oncology, Northwestern University, Chicago, IL 60611, USA; iuliia.topchu@northwestern.edu (I.T.); eroshchina@capefearvalley.com (E.R.); 2 Institute of Fundamental Medicine and Biology, Kazan Federal University, 420008 Kazan, Russia; 3 Cancer Signaling and Microenvironment Program, Fox Chase Cancer Center, Philadelphia, PA 19111, USA; igor.bychkov@fccc.edu (I.B.); petr.makhov@fccc.edu (P.M.); 4 O’Neil Comprehensive Cancer Center at University of Alabama at Birmingham, Department of Medicine, Section of Hematology/Oncology, Heersink School of Medicine, WTI, Room 510D, 1824 6th Ave S, Birmingham, AL 35233, USA

## Additional Affiliation

In the published publication [1], there was an error regarding the affiliation for “Iuliia Topchu”. In addition to the affiliation, “2” should be “Institute of Fundamental Medicine and Biology, Kazan Federal University, 420008 Kazan, Russia”. The authors apologize for any inconvenience caused and state that the scientific conclusions are unaffected. This correction was approved by the Academic Editor. The original publication has also been updated.

## References

[1] Topchu I., Bychkov I., Roshchina E., Makhov P., Boumber Y. (2024). PIP4K2B Protein Regulation by NSD1 in HPV-Negative Head and Neck Squamous Cell Carcinoma. Cancers.

