# Supplementary material for: PIP4K2B Protein Regulation by NSD1 in HPV-Negative Head and Neck Squamous Cell Carcinoma
Source: Cancers (Basel). 2024 Mar 17;16(6):1180. doi: 10.3390/cancers16061180 (PMC10968846; doi:10.3390/cancers16061180)

Figure 1B

JHU 011

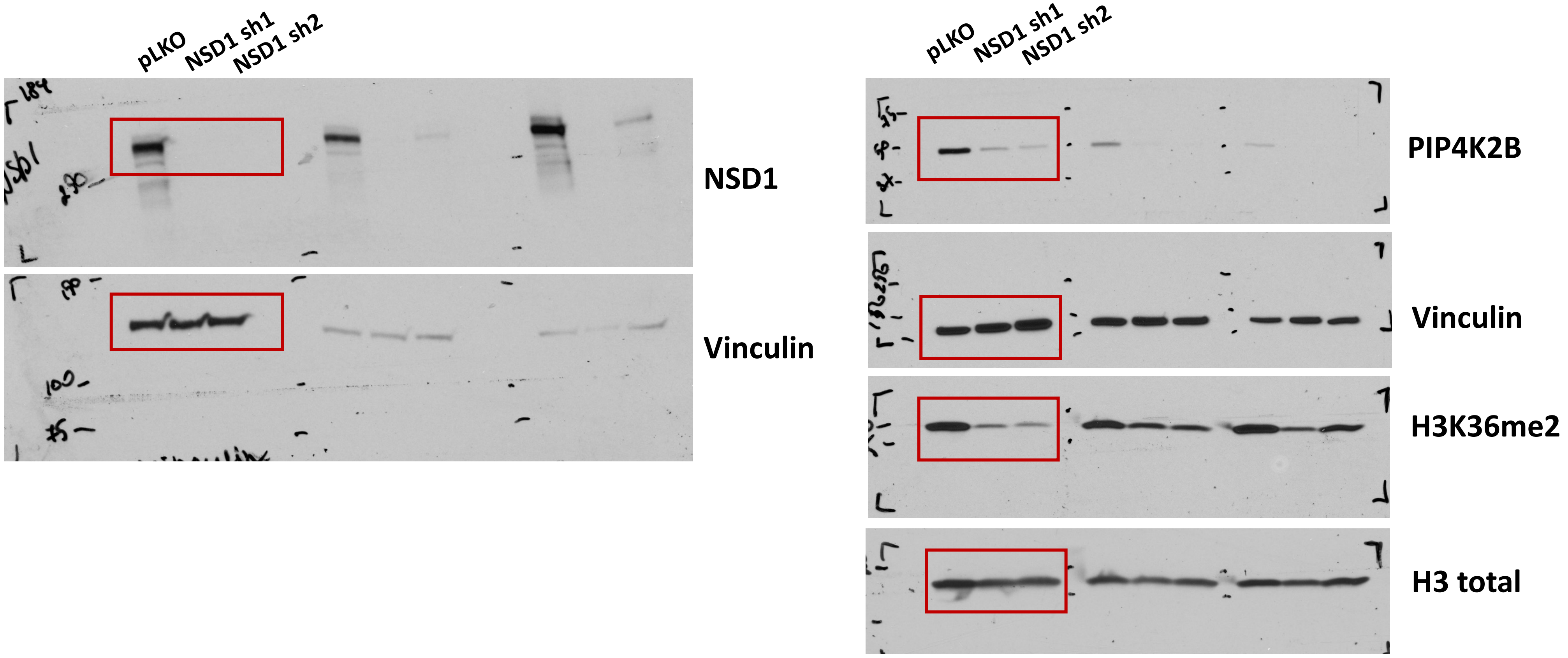

Figure 1B

Cal27

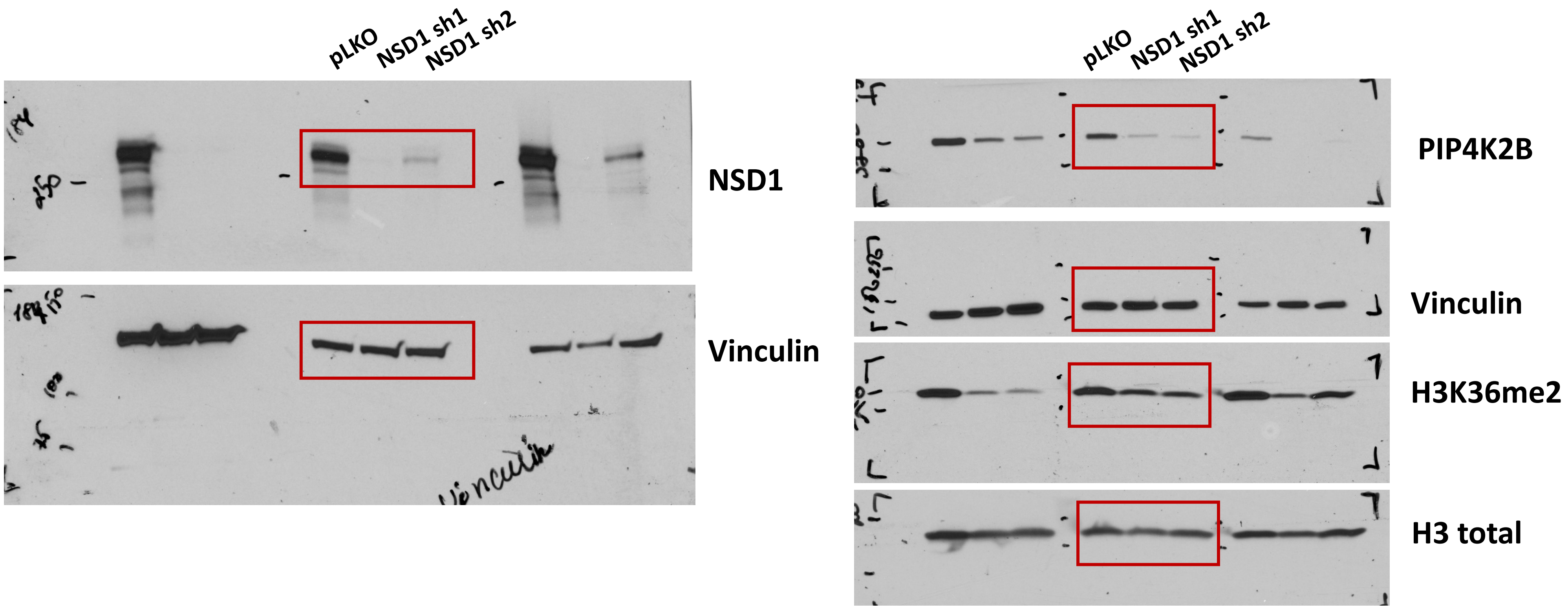

Figure 1B

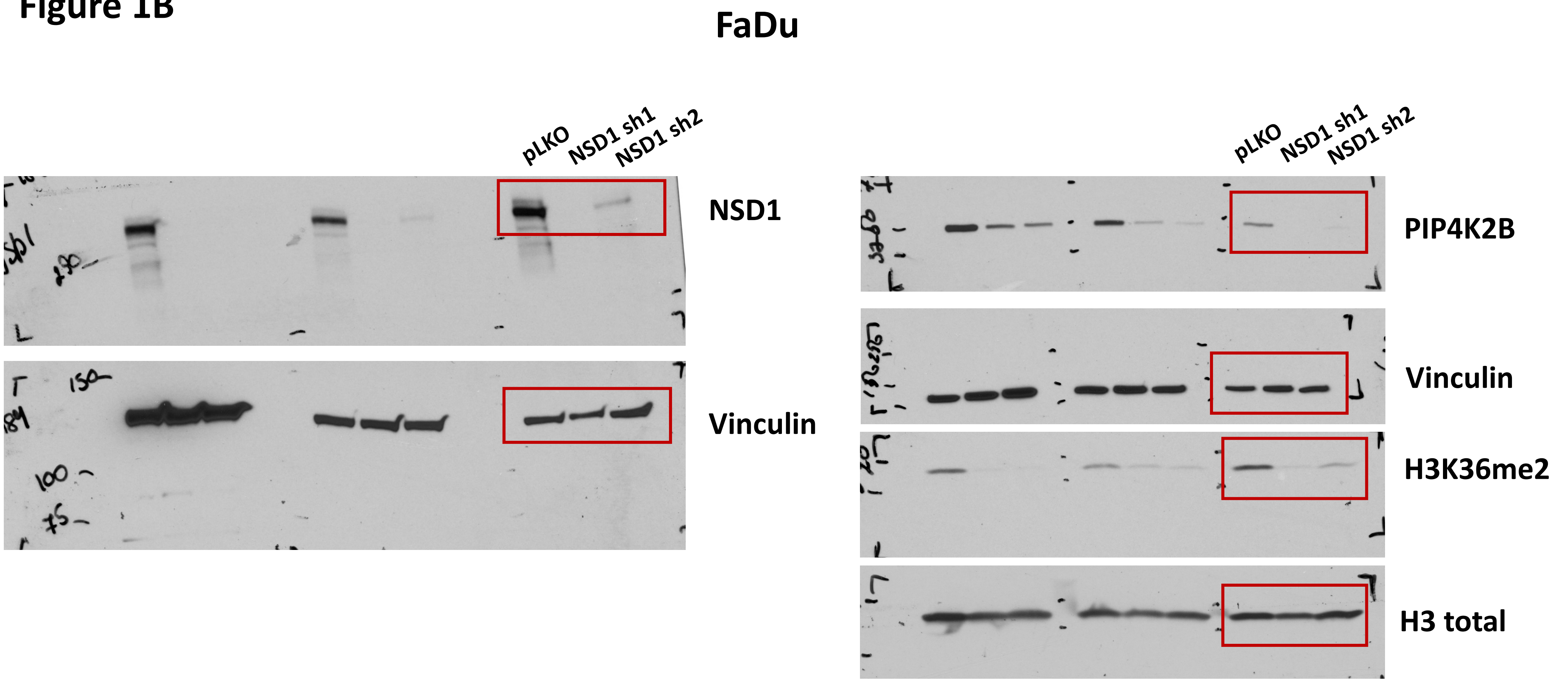
FaDu
pLKO
NSD1 sh1
NSD1 sh2
NSD1
Vinculin
pLKO
NSD1 sh1
NSD1 sh2
PIP4K2B
Vinculin
H3K36me2
H3 total

Figure 1D

JHU 011

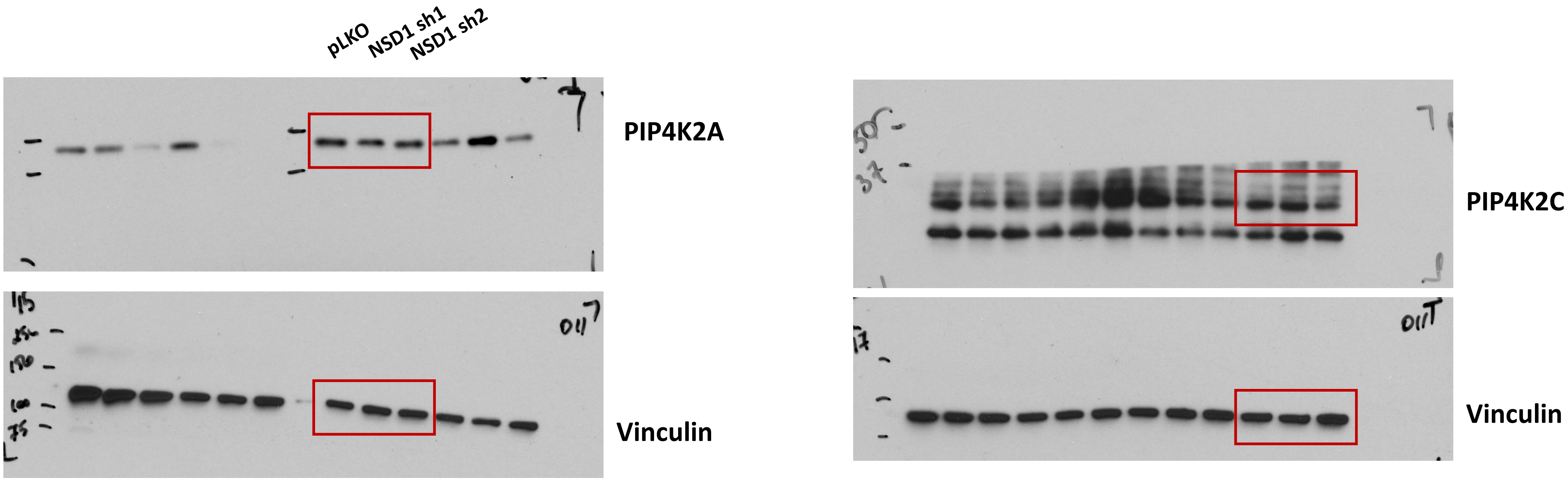

Figure 1D

Cal27

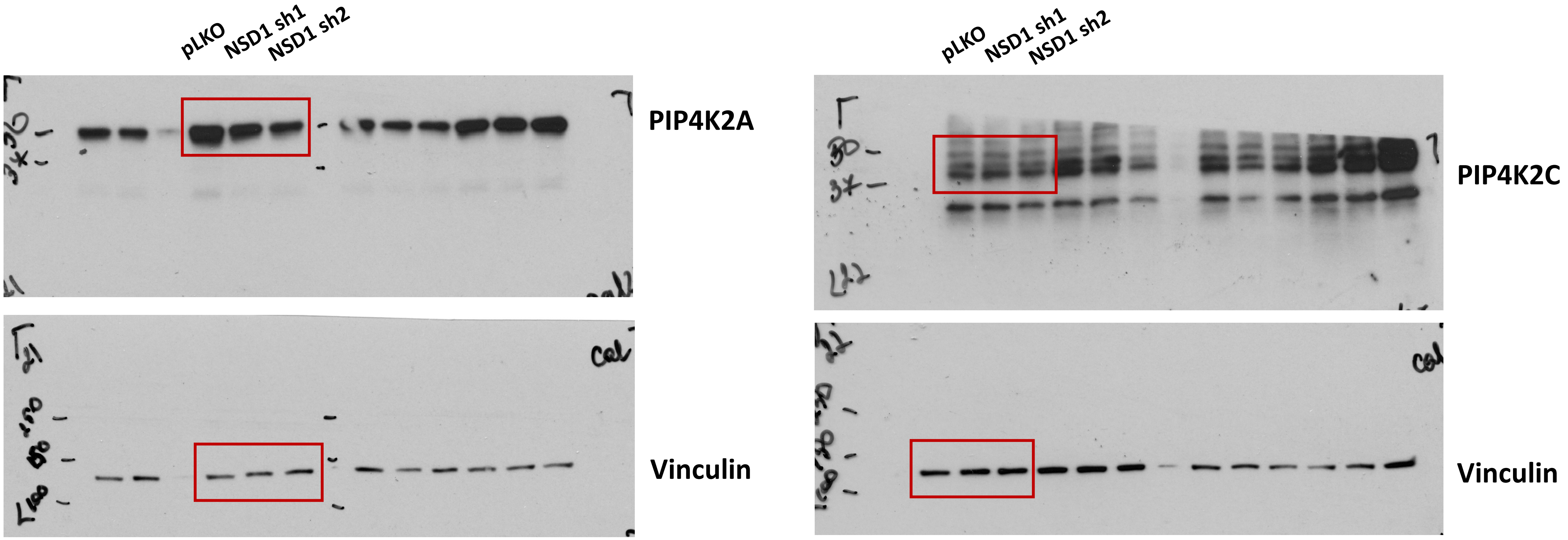

Figure 1D

FaDu

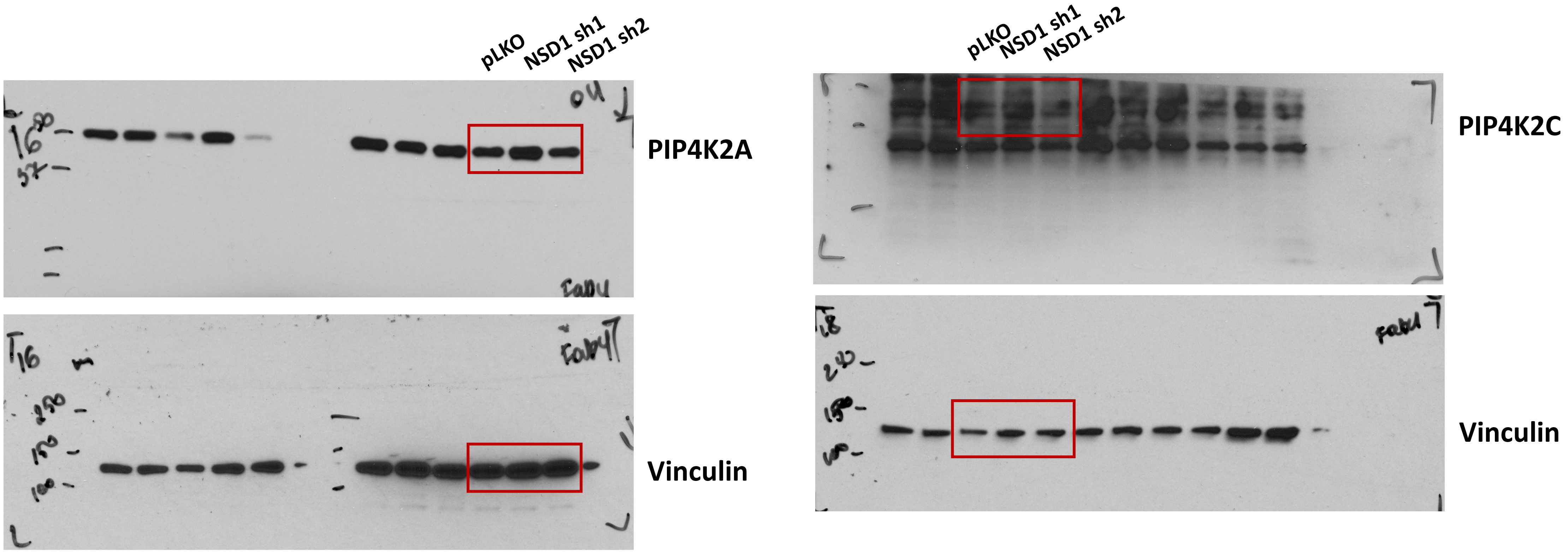

Figure 3A

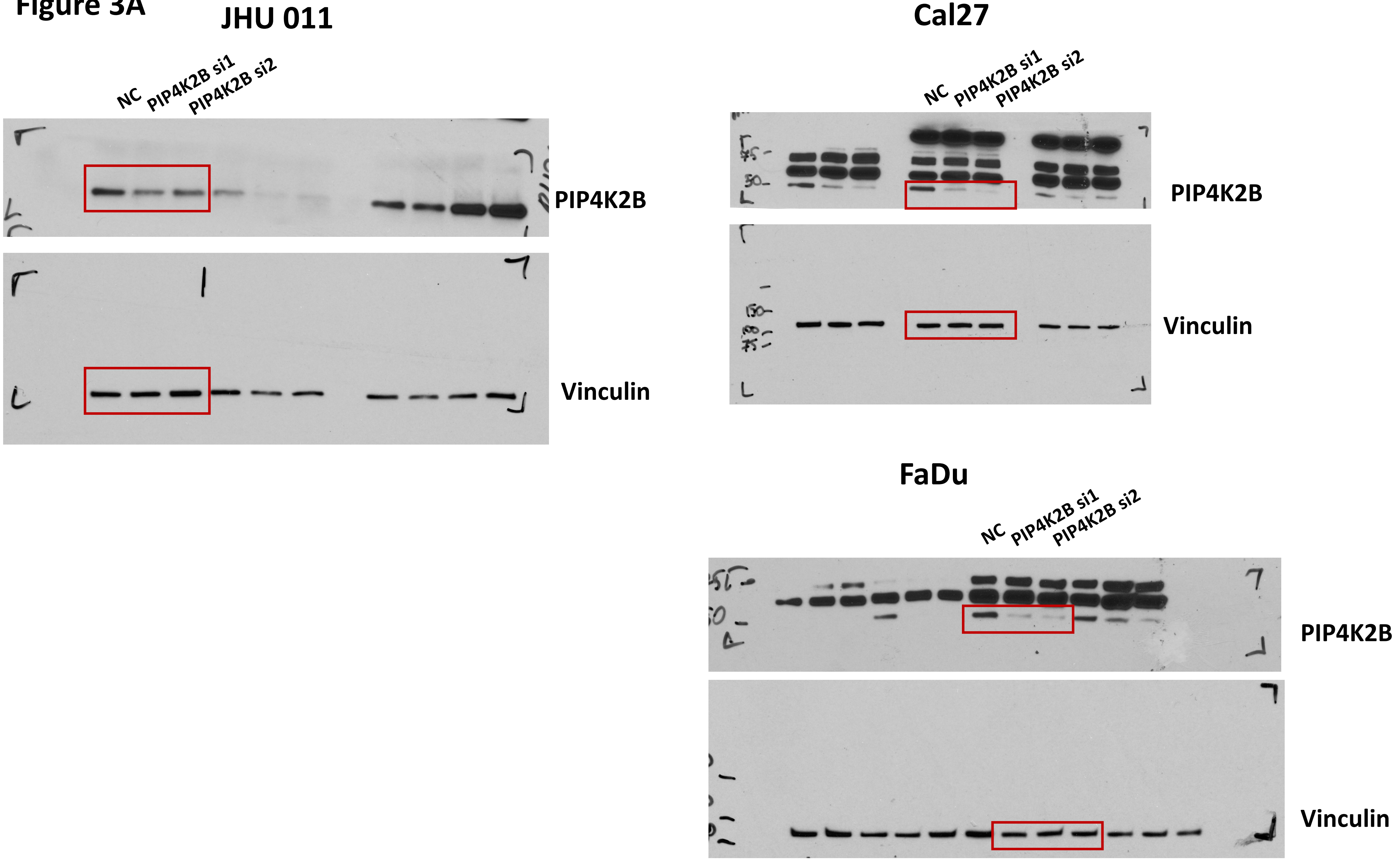

JHU 011
NC
PIP4K2B si1
PIP4K2B si2
PIP4K2B
Vinculin
Cal27
NC
PIP4K2B si1
PIP4K2B si2
PIP4K2B
Vinculin
FaDu
NC
PIP4K2B si1
PIP4K2B si2
PIP4K2B
Vinculin

Figure 3D

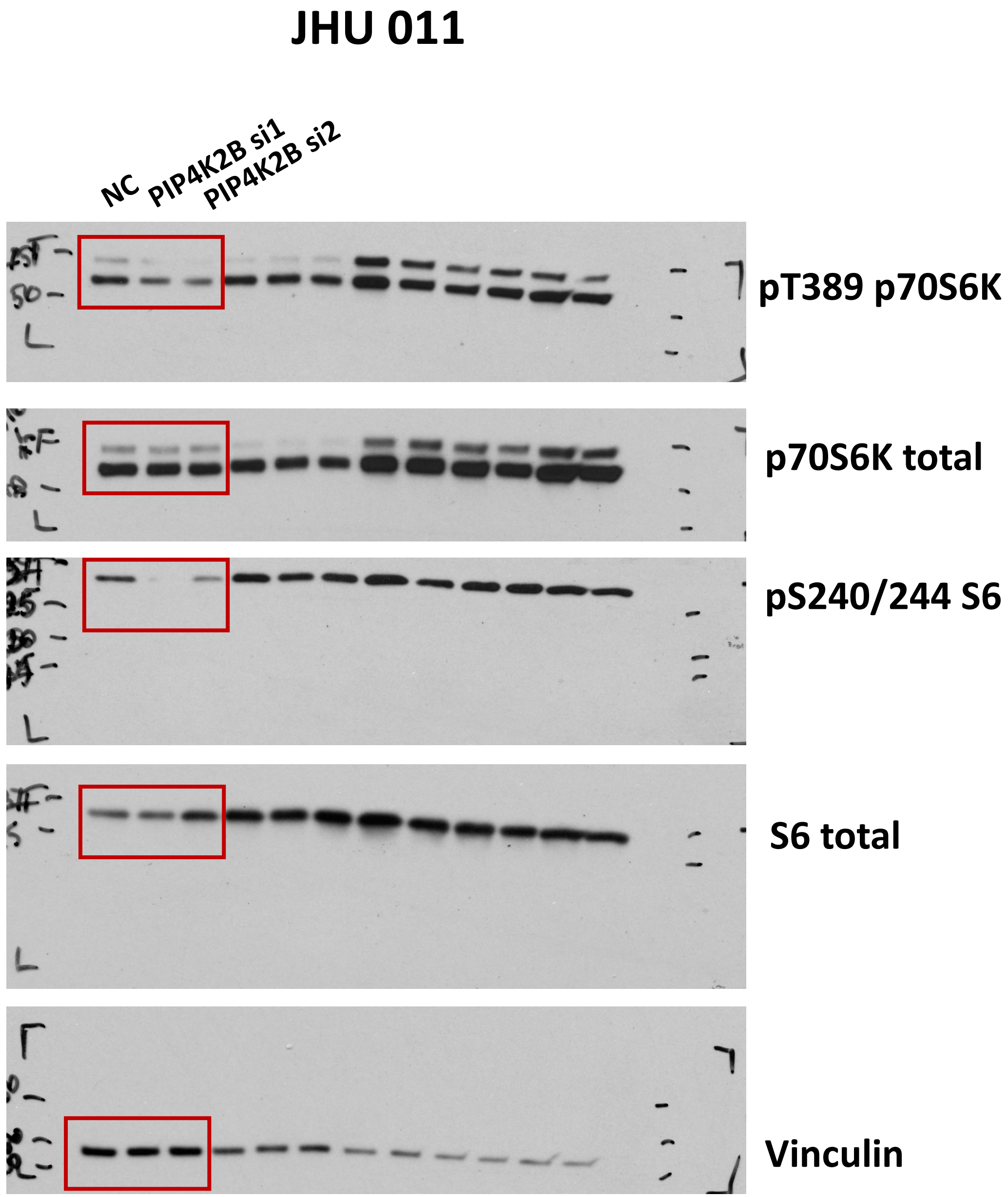
JHU 011
NC
PIP4K2B si1
PIP4K2B si2
pT389 p70S6K
p70S6K total
pS240/244 S6
S6 total
Vinculin

Figure 3D

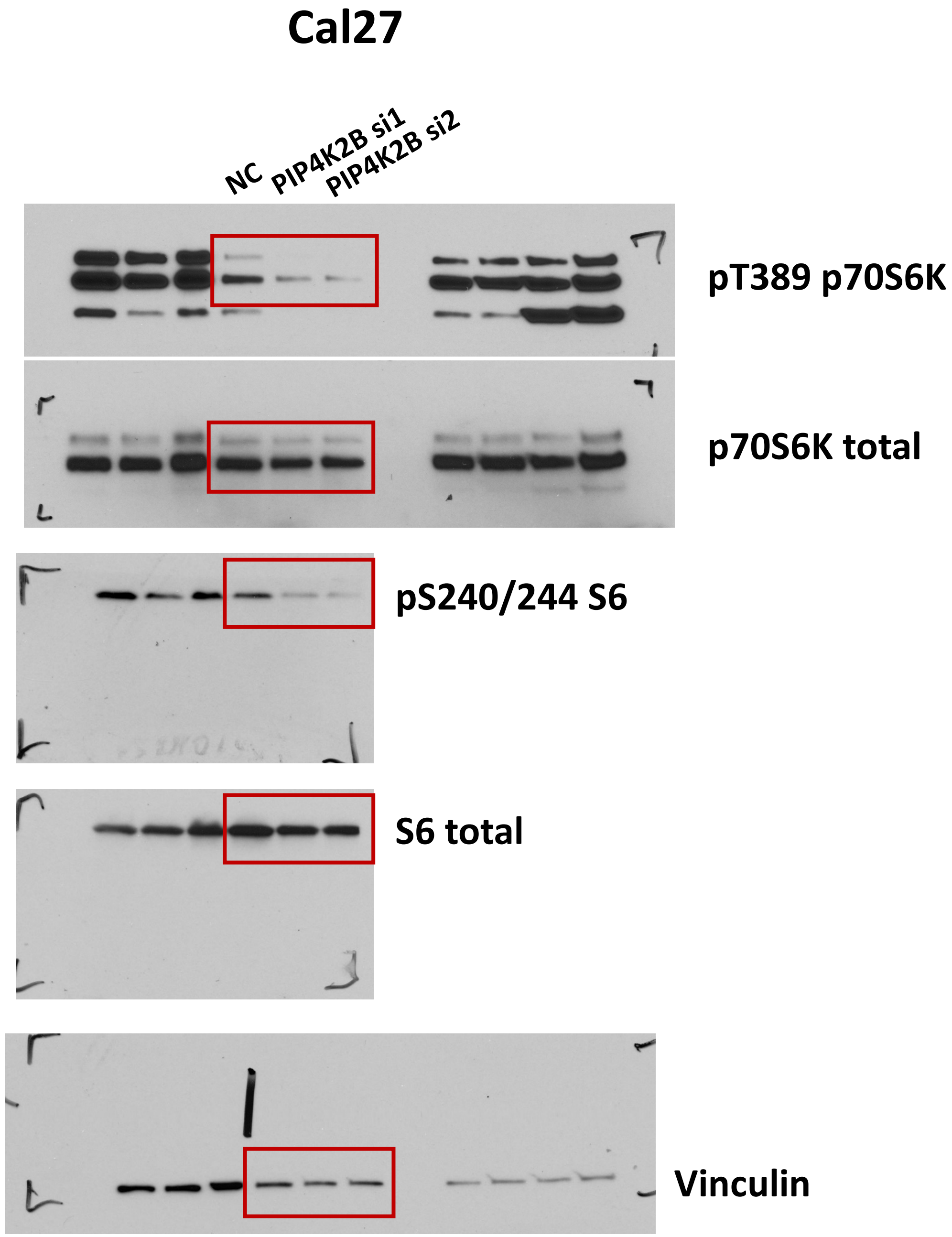

Cal27
NC
PIP4K2B si1
PIP4K2B si2
pT389 p70S6K
p70S6K total
pS240/244 S6
S6 total
Vinculin

Figure 3D

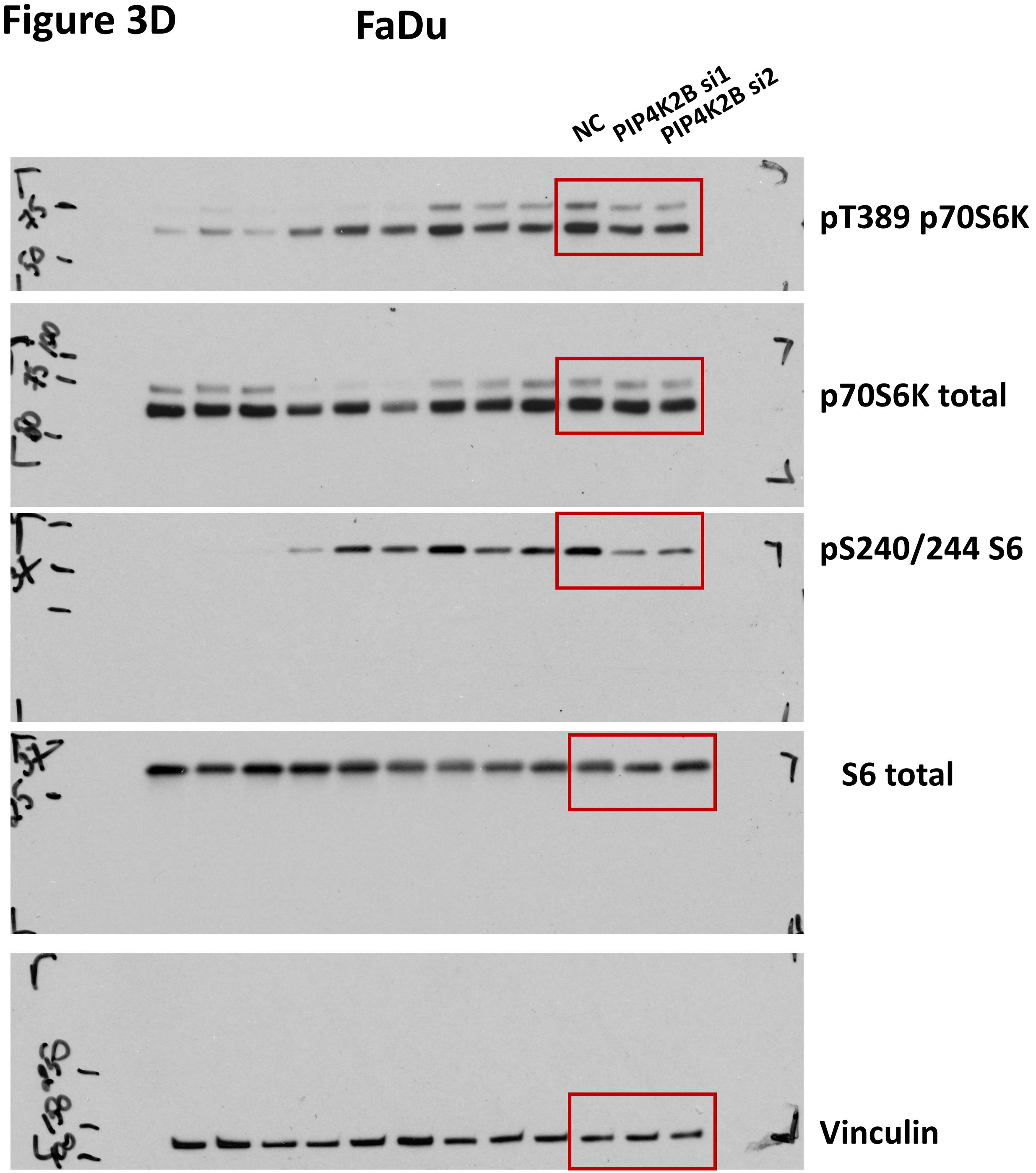

FaDu
NC
PIP4K2B si1
PIP4K2B si2
pT389 p70S6K
p70S6K total
pS240/244 S6
S6 total
Vinculin

Figure 4A

JHU 011

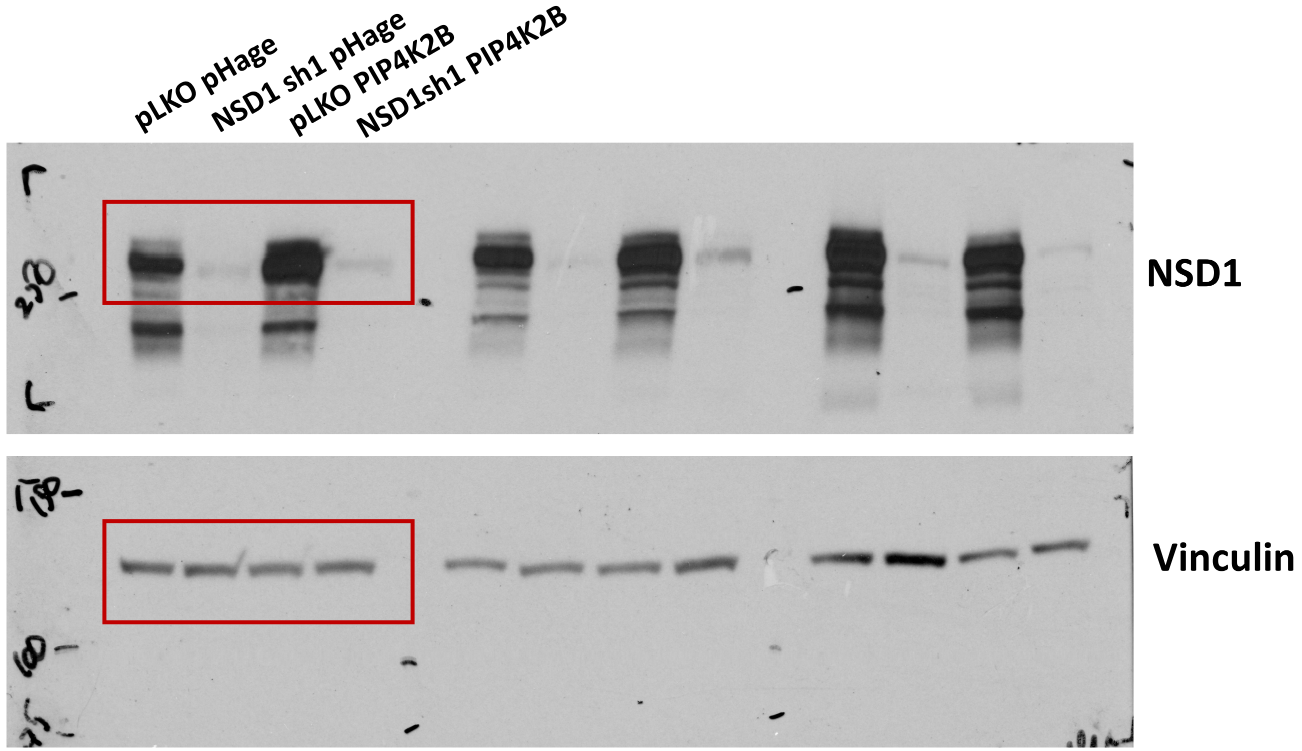

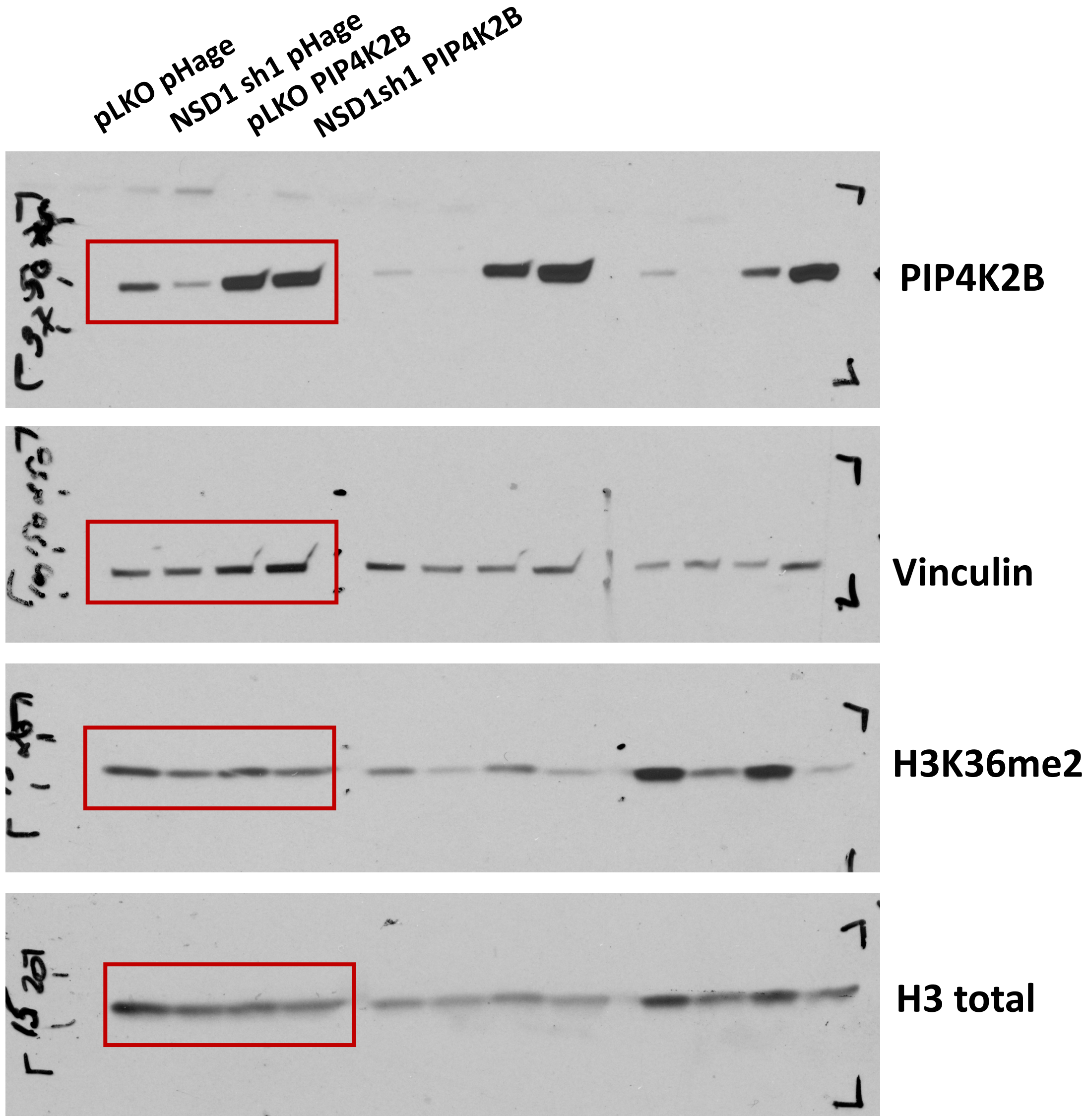

Figure 4A

Cal27

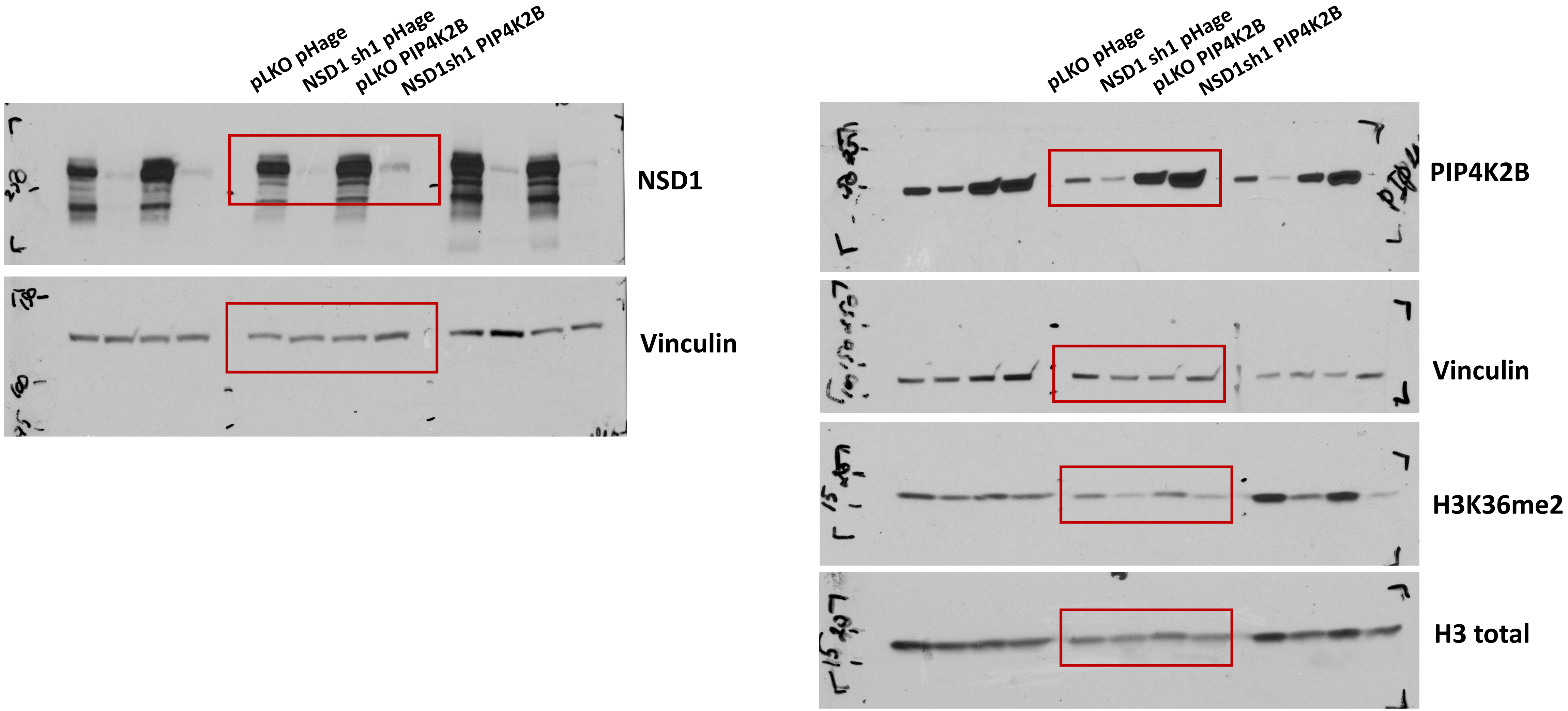

Figure 4A

FaDu

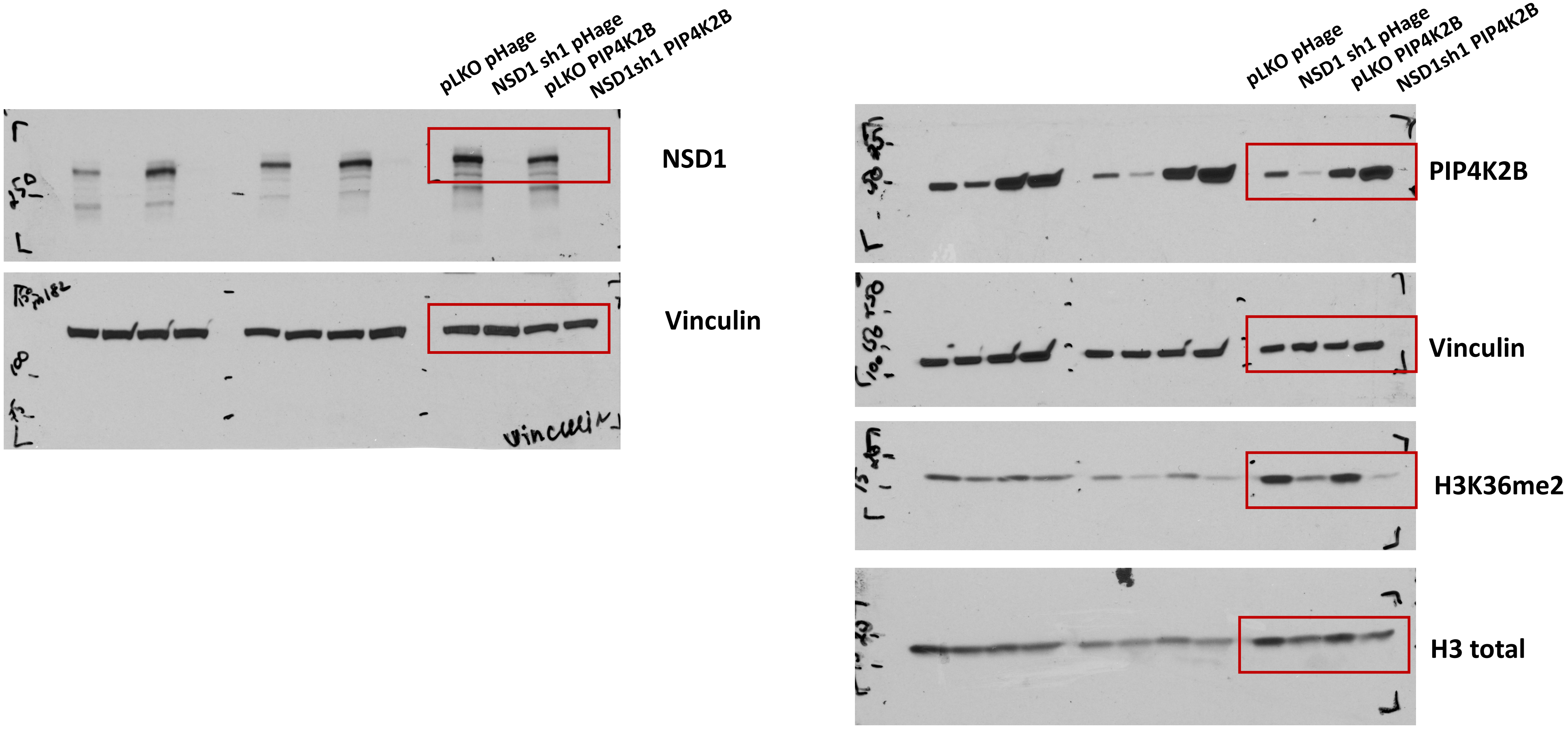

Figure 4D

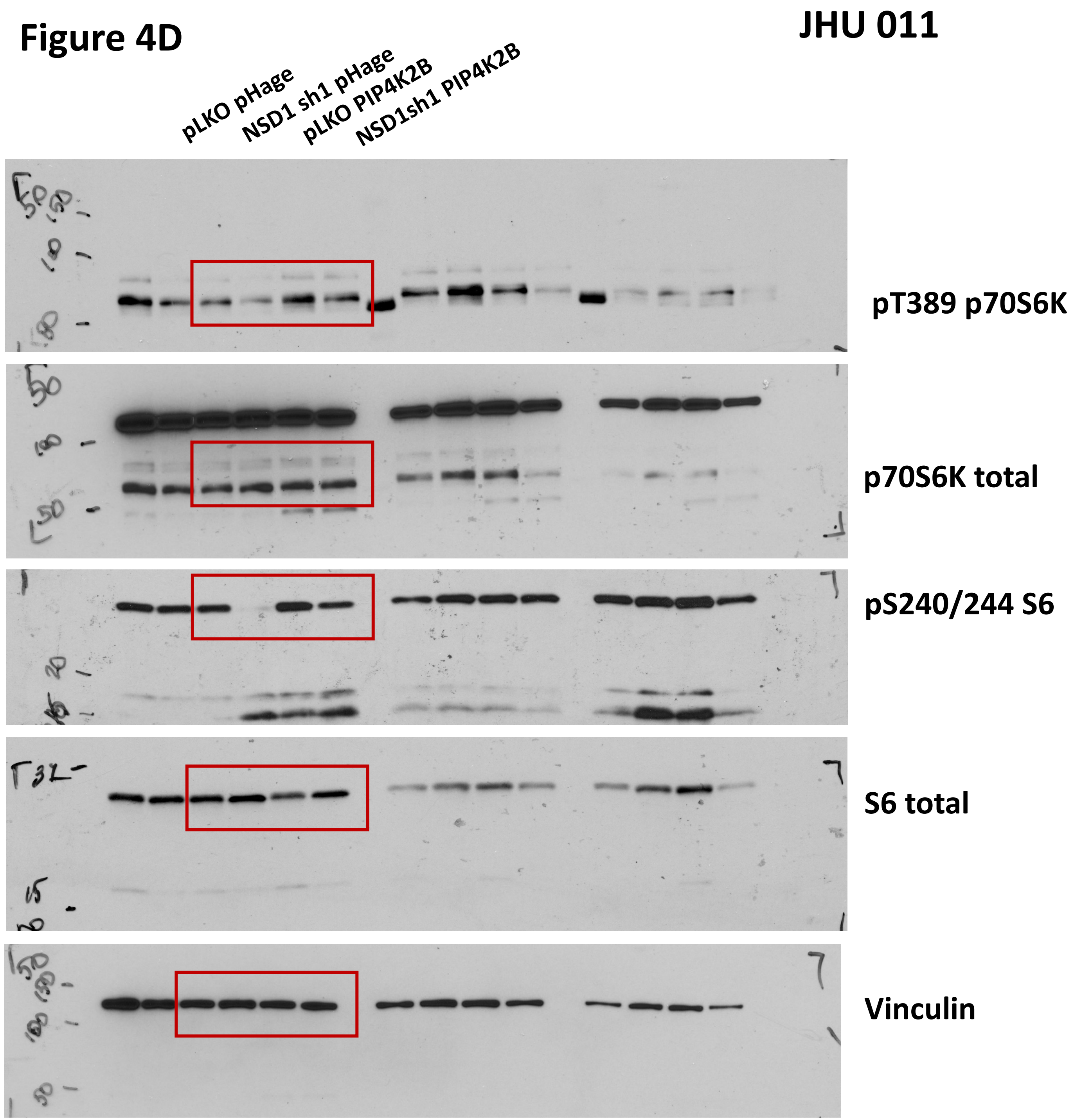
JHU 011
pLKO pHage
NSD1 sh1 pHage
pLKO PIP4K2B
NSD1sh1 PIP4K2B
pT389 p70S6K
p70S6K total
pS240/244 S6
S6 total
Vinculin

Figure 4D

Cal27

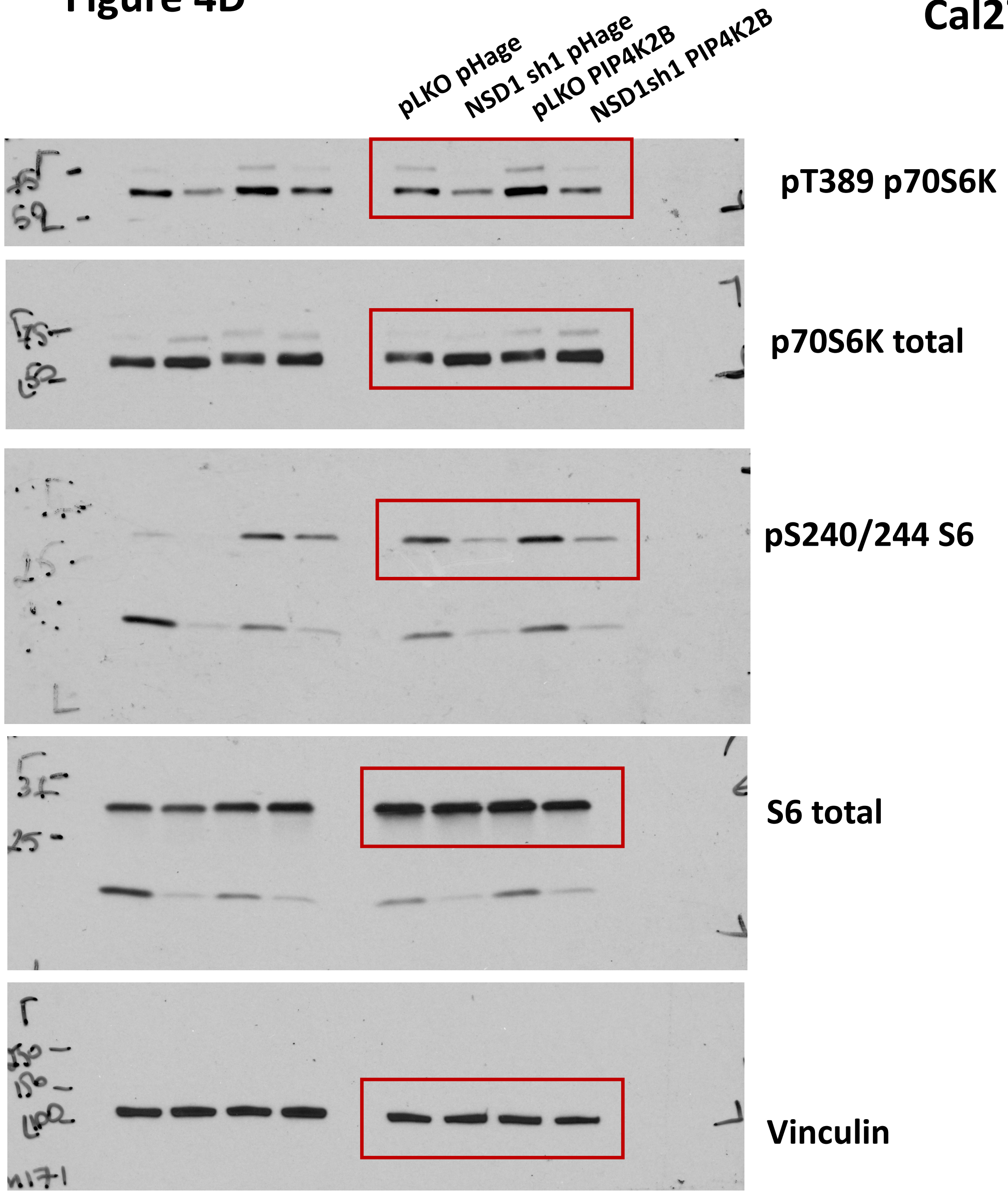

Figure 4D

FaDu

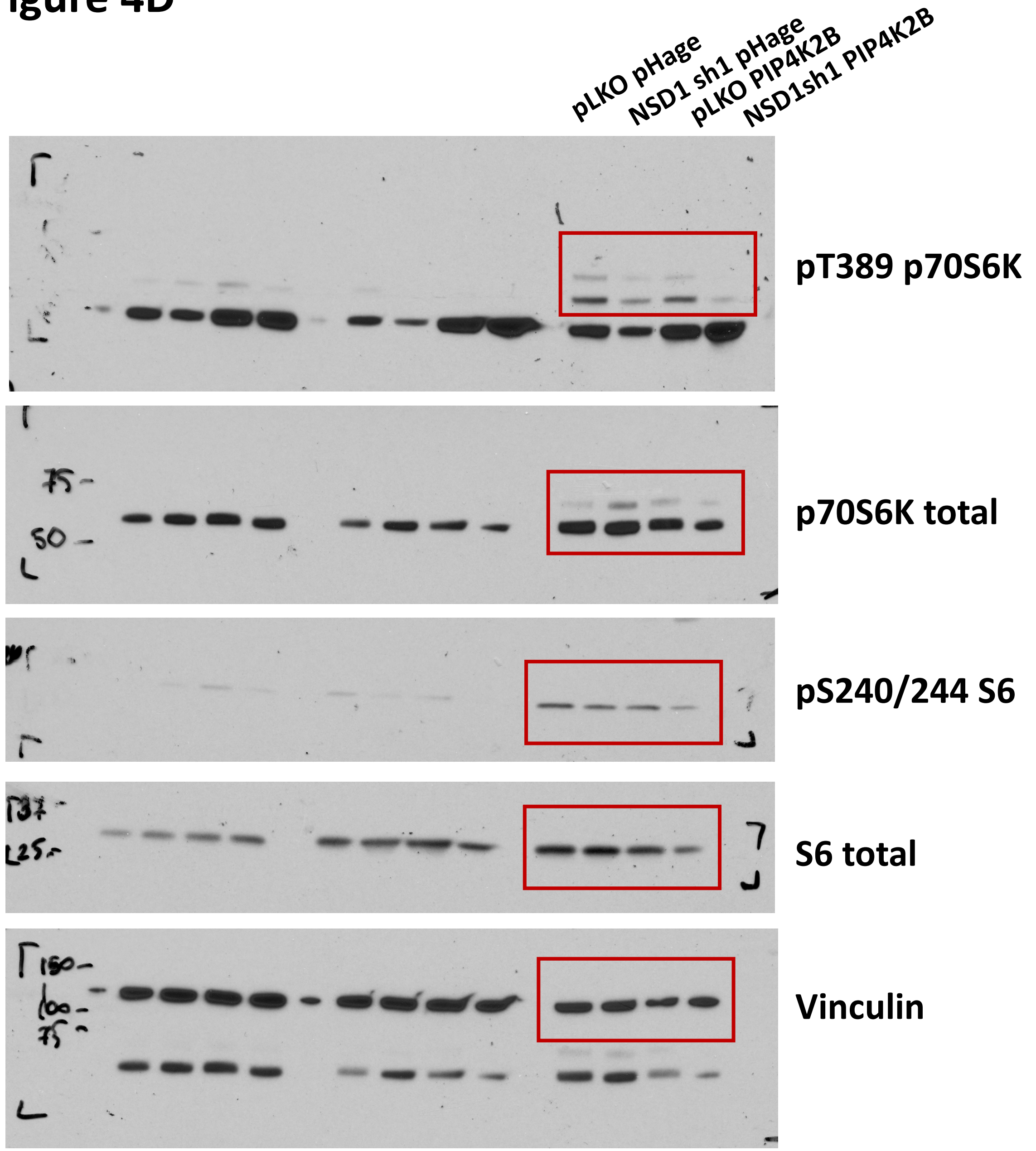

Supplement: Supplementary file 1 [file cancers-16-01180-s001.zip › File S1. Western blot original data.pdf]
