# Supplementary material for: PIP4K2B Protein Regulation by NSD1 in HPV-Negative Head and Neck Squamous Cell Carcinoma
Source: Cancers (Basel). 2024 Mar 17;16(6):1180. doi: 10.3390/cancers16061180 (PMC10968846; doi:10.3390/cancers16061180)
Supplement: Supplementary file 1 [file cancers-16-01180-s001.zip › Supplementary Figures.pdf]

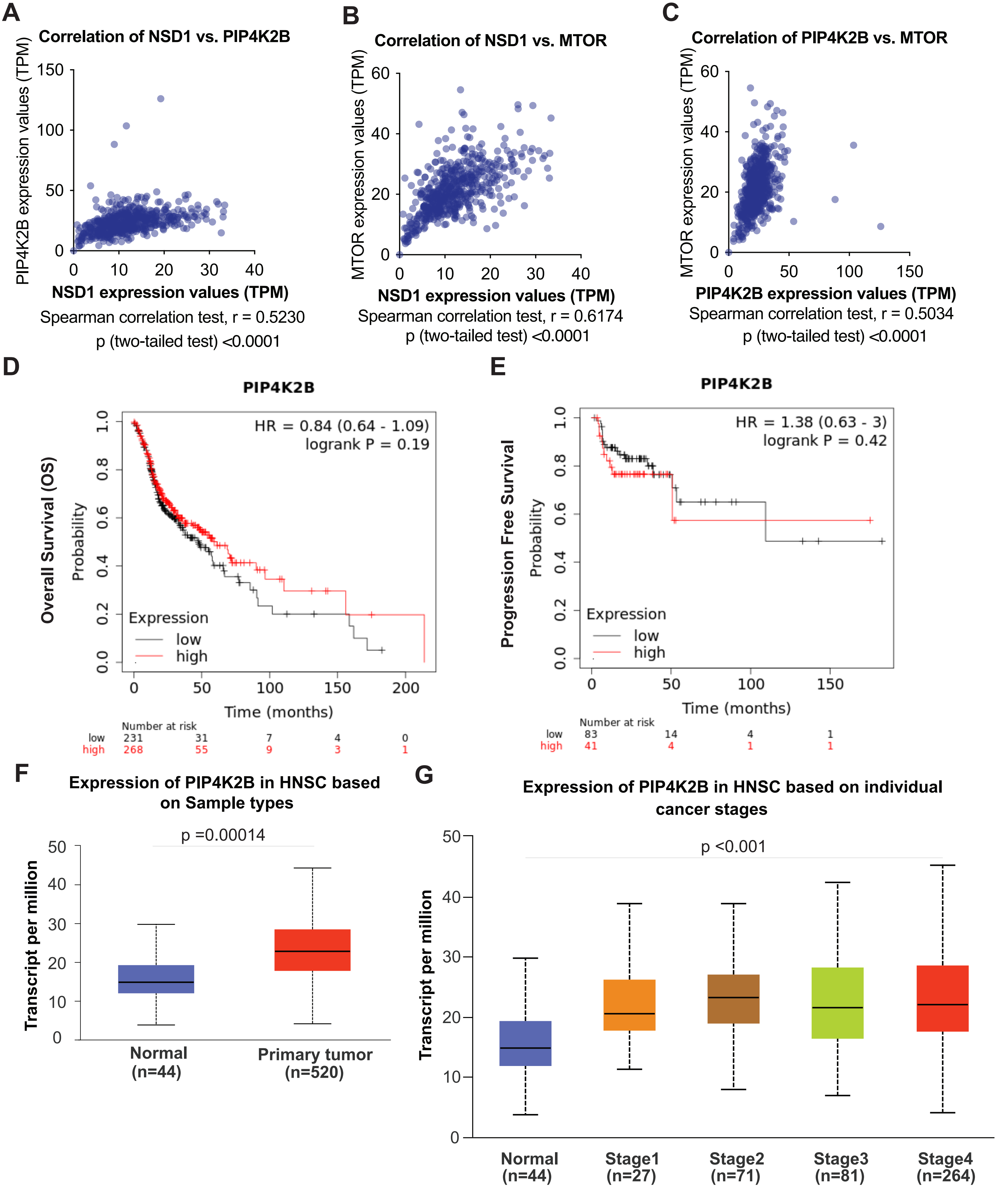

Supplementary Figure S1. Analysis of the TCGA HNSCC dataset. (A) NSD1 and PIP4K2B (n = 521), (B) NSD1 and MTOR (n = 521), (C) PIP4K2B and MTOR (n = 521) mRNA expression correlation in human tumor TCGA samples visualized by UALCAN database. (D, E) Kaplan–Meier analysis of overall (D) and progression-free (E) survival in TCGA RNA-seq HNSCC dataset (n=500), PIP4K2B mRNA expression automatically split by optimal cutoff value (low vs. high). (F) Analysis of PIP4K2B mRNA expression in HNSCC tissues and normal tissues, and (G) classification by tumor stages using the TCGA dataset, visualized by UALCAN database.

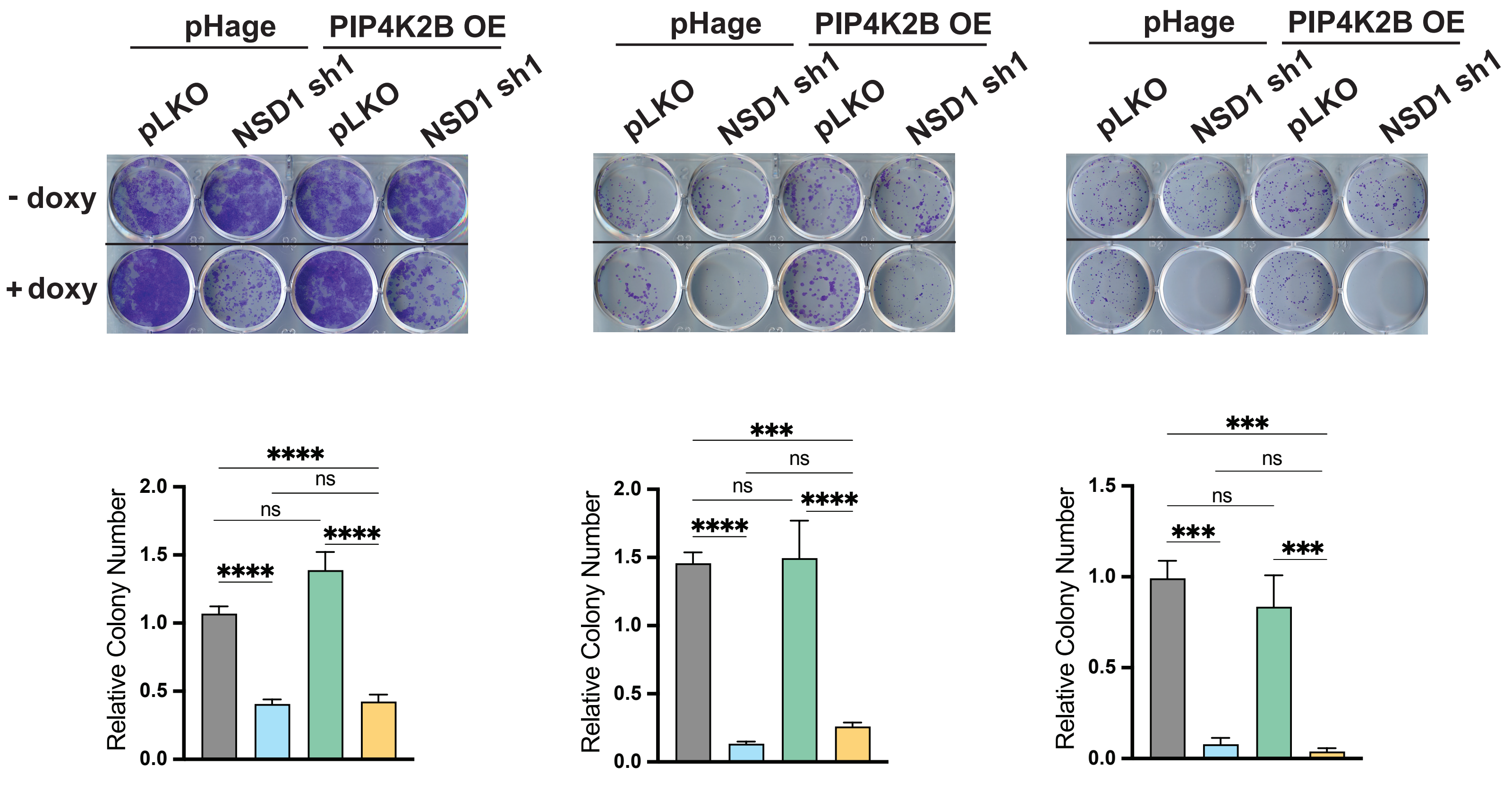

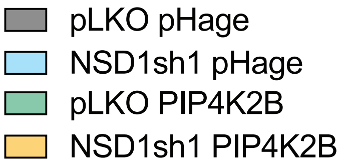

Supplementary Figure S2. Colony formation assay with PIP4K2B protein overexpression along with NSD1 knockdown. Representative images of colony formation assay and quantification of the relative colony numbers in JHU 011, Cal27, FaDu cell lines with PIP4K2B protein overexpression and NSD1 knockdown. Statistical significance is determined by the Mann-Whitney test, pairwise comparing every group to other.Data are performed from at least 3 independent biological repeats. The error bars are presented as mean ± SEM. ns – not significant, *p<0.05, **p<0.01, ***p<0.001, and ****p<0.0001.
