# Supplementary material for: PIP4K2B Protein Regulation by NSD1 in HPV-Negative Head and Neck Squamous Cell Carcinoma
Source: Cancers (Basel). 2024 Mar 17;16(6):1180. doi: 10.3390/cancers16061180 (PMC10968846; doi:10.3390/cancers16061180)
Supplement: Supplementary file 1 [file cancers-16-01180-s001.zip › Supplementary tables.pdf]

Supplementary Table S1. shRNA oligos

NSD1 shRNA oligo 1 fw	CCGG CCGAGACGTCTCAGGTTAATCCTCGAGGATTAACCTGAGACGTCTCGGTTTTT
NSD1 shRNA oligo 1 rev	AATT AAAAACCGAGACGTCTCAGGTTAATCCTCGAGGATTAACCTGAGACGTCTCGG
NSD1 shRNA oligo 2 fw	CCGG AGGAGTGGATGGGACATATAACTCGAGTTATATGTCCCATCCACTCCTTTTT
NSD1 shRNA oligo 2 rev	AATT AAAAAGGAGTGGATGGGACATATAACTCGAGTTATATGTCCCATCCACTCCT

Table S2. PCR primer sequences for ChIP-assay

Gene symbol	Sequence	Supplier
PIP4K2B (-1409)	5'-CCAGCCTGGGTGACAGAGCG-3'	Integrated DNA Technologies
	3'-CGCCCAGCTAATGTGCGGGT-5'	
PIP4K2B (-1009)	5'-CACCTGTGGAGTACCGTGTT-3'	Integrated DNA Technologies
	3'-GAGCTGGTTACCGTGTGGTT-5'	
PIP4K2B (-1)	5'- CATGTCGTCCAACTGCACCAG -3	Integrated DNA Technologies
	3'-GAGCTGGTTACCGTGTGGTT-5'	
PIP4K2B (+29596)	5'- GCAGCCTCCTGATTGGGGCA -3'	Integrated DNA Technologies
	3'- CTCCCGATCCCCGACCCCAT -5'	

Supplementary Table S3. siRNA sequences

Gene symbol		Supplier	Catalog number
PIP4K2B si1	5`- CAUGUUACGAUGAAUCUCCAACCTG - 3`	Integrated DNA Technologies	#309482066 (hs.Ri.PIP4K2B.13.1)
	3`-ACGUACAAUGCUACUUAGAGGUUGGAC -5`		
PIP4K2B si2	5`- AUUGAUGAUCAGGAUUACCAGAATT -3`	Integrated DNA Technologies	#309482069 (s.Ri.PIP4K2B.13.2)
	3`- CUUAACUACUAGUCCUAAUGGUCUUAA-5`		
